# Interobserver and intraobserver variability of fetal and maternal Doppler measurements: systematic review and meta‐analysis

**DOI:** 10.1002/uog.70287

**Published:** 2026-08-08

**Authors:** L. I. Prins, N. El Guili, C. A. Naaktgeboren, N. P. Denswil, R. J. Burger, S. J. Gordijn, W. Ganzevoort

**Affiliations:** ^1^ Department of Obstetrics and Gynecology Amsterdam UMC location University of Amsterdam Amsterdam The Netherlands; ^2^ Amsterdam Reproduction and Development Amsterdam The Netherlands; ^3^ Department of Epidemiology Amsterdam UMC location University of Amsterdam Amsterdam The Netherlands; ^4^ Department of Medical Library Amsterdam UMC location University of Amsterdam Amsterdam The Netherlands; ^5^ Department of Obstetrics and Gynecology University Medical Center Groningen Groningen The Netherlands

**Keywords:** Doppler ultrasonography, observer variation, reproducibility of results

## Abstract

**Objectives:**

To evaluate the variability and reproducibility of umbilical artery (UA), fetal middle cerebral artery (MCA) and uterine artery (UtA) Doppler ultrasound measurements in pregnancy.

**Methods:**

A systematic search of MEDLINE, EMBASE and the Cochrane Library (CENTRAL) was conducted from inception to 17 June 2024. Studies were included if they reported intra‐ or interobserver variability of UA, MCA or UtA Doppler ultrasound measurements in singleton or multiple pregnancies, using the intraclass correlation coefficient (ICC), concordance correlation coefficient, Cohen's kappa or limits of agreement. Data extraction was performed using standardized data‐extraction forms. A meta‐analysis was conducted using a random‐effects model to account for heterogeneity, and variability estimates were pooled using Fisher's *Z*‐transformation. Reproducibility was categorized according to the True Reproducibility of Ultrasound Techniques (TRUST) criteria.

**Results:**

In total, 2426 records were screened. Of these, 26 studies including 2457 patients met the inclusion criteria; eight studies described UA Doppler, 11 described MCA Doppler and 11 described UtA Doppler results; two of the studies described cerebroplacental ratio results. The pooled intraobserver ICC for UA pulsatility index (PI) was 0.88 (95% CI, 0.77–0.94) and the pooled interobserver ICC was 0.68 (95% CI, 0.55–0.79). For MCA‐PI, these values were 0.85 (95% CI, 0.69–0.93) and 0.80 (95% CI, 0.62–0.90), respectively, and for mean UtA‐PI they were 0.90 (95% CI, 0.85–0.93) and 0.84 (95% CI, 0.78–0.89), respectively. Findings across studies suggested generally poor‐to‐moderate reproducibility according to the strict TRUST criteria.

**Conclusions:**

Our results emphasize that there is room for improvement regarding the reproducibility of Doppler ultrasound in routine obstetric practice. By standardizing ultrasound measurement protocols, advancing technologies such as artificial‐intelligence‐supported measurement applications and training programs, as well as obtaining multiple measurements per session and performing quality audits when outliers are detected, the field could move towards more accurate and reliable fetal assessments. © 2026 The Author(s). *Ultrasound in Obstetrics & Gynecology* published by John Wiley & Sons Ltd on behalf of International Society of Ultrasound in Obstetrics and Gynecology.

## INTRODUCTION

Estimation of fetal wellbeing is a crucial component of prenatal care, aimed at determining the risks of adverse pregnancy outcomes^1^, ^2^. A widely used non‐invasive method to evaluate placental function and fetal wellbeing is Doppler ultrasonography. The vascular pulsatility indices (PIs) of the maternal uterine artery (UtA), the umbilical artery (UA), the fetal middle cerebral artery (MCA) and the ductus venosus (DV) have become particularly important parameters for predicting and detecting fetal compromise, especially in high‐risk pregnancies^3^. These measurements provide insights into placental (dys)function and the fetal hemodynamic compensatory mechanism for reduced metabolic and respiratory exchange^3^.

When performing Doppler ultrasound measurements, high reproducibility, defined as the ability to obtain consistent results under the same conditions, and low variability, defined as the degree of difference between repeated measurements, are crucial for the interpretation of fetal wellbeing assessments and thereby clinical decision‐making^4^, ^5^. On the one hand, inconsistent or unreliable results may lead to inappropriate clinical interventions, such as unnecessary (early) iatrogenic birth, while on the other hand, they may lead to withholding of essential interventions such as increased fetal monitoring or timely initiation of birth^1^, ^4^, ^6^.

The True Reproducibility of Ultrasound Techniques (TRUST) criteria were introduced in 2015 to standardize the interpretation of variability in ultrasound measurement techniques, including Doppler measurements^7^, ^8^. These criteria provide specific guidelines for performing, interpreting and reporting ultrasound measurements, including Doppler studies, focusing on inter‐ and intraobserver variability, in order to enhance their reproducibility across different settings and thereby their usefulness in guiding clinical management decisions^9^. The criteria are strict, because high reproducibility is important for clinical decision‐making.

The TRUST criteria have not been applied consistently in all settings. Ideally, they should be applied in all Doppler measurement studies to optimize reproducibility. However, many studies continue to use other methods to classify the variability of Doppler ultrasound results^10^, ^11^.

Bruin *et al*.^12^ recently reported that intraclass correlation coefficients (ICCs) for DV‐PI measurements vary considerably among different published studies. A similar review of the variability of Doppler measurements in the UA, MCA and UtA is lacking. Therefore, our aim was to evaluate the reproducibility of UA, MCA and UtA Doppler ultrasound measurements and of the calculated cerebroplacental ratio in pregnancy.

## METHODS

### Registration and protocol

The protocol for this systematic review was registered prospectively in the International Prospective Register of Systematic Reviews (PROSPERO) (https://www.crd.york.ac.uk/prospero/; registration number: CRD42024515971) in accordance with the Preferred Reporting Items for Systematic Review and Meta‐Analysis Protocols (PRISMA‐P)^13^.

### Eligibility

Studies including women with a singleton or multiple pregnancy at high or low risk for fetal growth restriction and for maternal hypertensive disorders were considered eligible for inclusion in our systematic review. Pregnancies of all gestational ages (GAs) were included. Studies were eligible for inclusion in the systematic review if they reported ICC, concordance correlation coefficient (CCC), Cohen's kappa or limits of agreement (LoA), including analyses based on categorical or abnormal Doppler values.

### Search strategy

To identify relevant studies for inclusion, we searched Ovid MEDLINE(R) ALL, Ovid EMBASE and the Cochrane Central Register of Controlled Trials (CENTRAL), from inception until 17 June 2024. No language restrictions were applied during the initial screening. Case studies, letters and animal studies were excluded. Other study types, including gray literature, were screened at the full‐text level when available. The search was performed by a medical information specialist (N.P.D.) and a medical doctor in obstetrics (L.I.P.). The following terms, including synonyms and closely related words, were used as index terms or free‐text words in the search: ‘ultrasonography Doppler’, ‘pregnancy’ and ‘inter‐ and intra‐observer variability’. Duplicate records were excluded by N.P.D. using DedupEndNote^14^. Full search details are given in Table S1.

### Study selection

Titles and abstracts were screened independently by two researchers (L.I.P. and a medical student, N.e.G.), using the Rayyan application^15^. Studies that met the inclusion criteria underwent full‐text screening by the same two authors. Any discrepancies during the screening process were resolved by discussion until consensus was reached. Studies were eligible if they met the following criteria: (1) population: they were conducted in pregnant patients; (2) intervention: multiple Doppler ultrasound measurements were performed in one or more of the UA, MCA and UtA, in order to assess the intra‐ or interobserver variability; (3) outcome: ICC, CCC, Cohen's kappa or LoA were reported.

### Data extraction

Data were extracted from the included studies using a data‐extraction form developed and tested by two researchers (L.I.P., N.e.G.). Data extraction was carried out twice by L.I.P. Any discrepancies were resolved by discussion with N.e.G. until consensus was reached. Extracted data included both data on Doppler‐specific measurements, including intra‐ and interobserver ICC and CCC, Cohen's kappa and LoA values, as well as numerous study characteristics, including the clinical setting, number of included patients, population type, maternal age, body mass index (BMI) and GA at assessment (mean/median and range). Ultrasound‐related factors were also extracted, including the vessel examined, the scanning approach (transabdominal or transvaginal), the type of ultrasound machine used, the level of sonographer experience, the method of blinding and interpretation of the results by the authors.

### Risk‐of‐bias and applicability assessment

For the risk‐of‐bias analysis, two researchers (L.I.P. and an epidemiologist and midwife in training, C.A.N.) independently evaluated the included studies using the standardized COSMIN risk‐of‐bias tool^16^. Any discrepancies found during the evaluation process were resolved through discussion. An overview of the risk‐of‐bias analysis is presented in Figure S1.

### Synthesis of evidence

We performed a meta‐analysis to pool the reported variability estimates across the included studies. In studies reporting ICCs, we applied a Fisher's *Z*‐transformation to stabilize variance prior to pooling the estimates. Variance of the ICC values was calculated using the sample‐size method^17^. The pooled Fisher's *Z‐*value was then back‐transformed to present a combined ICC estimate. The same strategy was applied for CCC values. For studies reporting Cohen's kappa statistics or LoA values, no transformation was necessary and the values were pooled directly. We used the TRUST criteria to classify reproducibility outcomes (Table 1)^7^.

**Table 1:** Interpretation of True Reproducibility of Ultrasound Techniques (TRUST) criteria*

ICC/CCC	Cohen's kappa	LoA (%)†	Interpretation
< 0.70	< 0.20	> 50	Very poor
0.70–0.90	0.21–0.40	20–50	Poor
> 0.90–0.95	0.41–0.60	10 to < 20	Moderate
> 0.95–0.99	0.61–0.80	5 to < 10	Good
> 0.99	≥ 0.81	< 5	Very good

* Based on Martins and Nastri^7^ and Coelho Neto *et al*.^8^.

† Limits of agreement (LoA) results are given as an interval; half the width of this interval is used when comparing results with the TRUST cut‐off values. CCC, concordance correlation coefficient; ICC, intraclass correlation coefficient.

### Heterogeneity assessment

Heterogeneity among the studies was evaluated using the Cochran's Q statistic and the *I* ^2^ statistic, which estimates the percentage of total variation across studies due to heterogeneity rather than chance. A random‐effects model was used to account for potential heterogeneity among the studies, following the DerSimonian and Laird method^18^.

### Sensitivity analysis

The primary analysis included all eligible studies, regardless of their risk‐of‐bias rating. To examine the robustness of our pooled estimates, we conducted a sensitivity analysis, excluding studies with a high risk of bias, those defined by COSMIN criteria as ‘inadequate’, or those having outliers for variability estimates. Furthermore, the sensitivity analyses were performed on data obtained using more recent ultrasound machines, including studies from 2015 onwards. The results of the sensitivity analysis were compared with those of the main meta‐analysis to assess the impact of study quality and statistical heterogeneity on the overall conclusions.

## RESULTS

### Study selection

**Figure 1:**
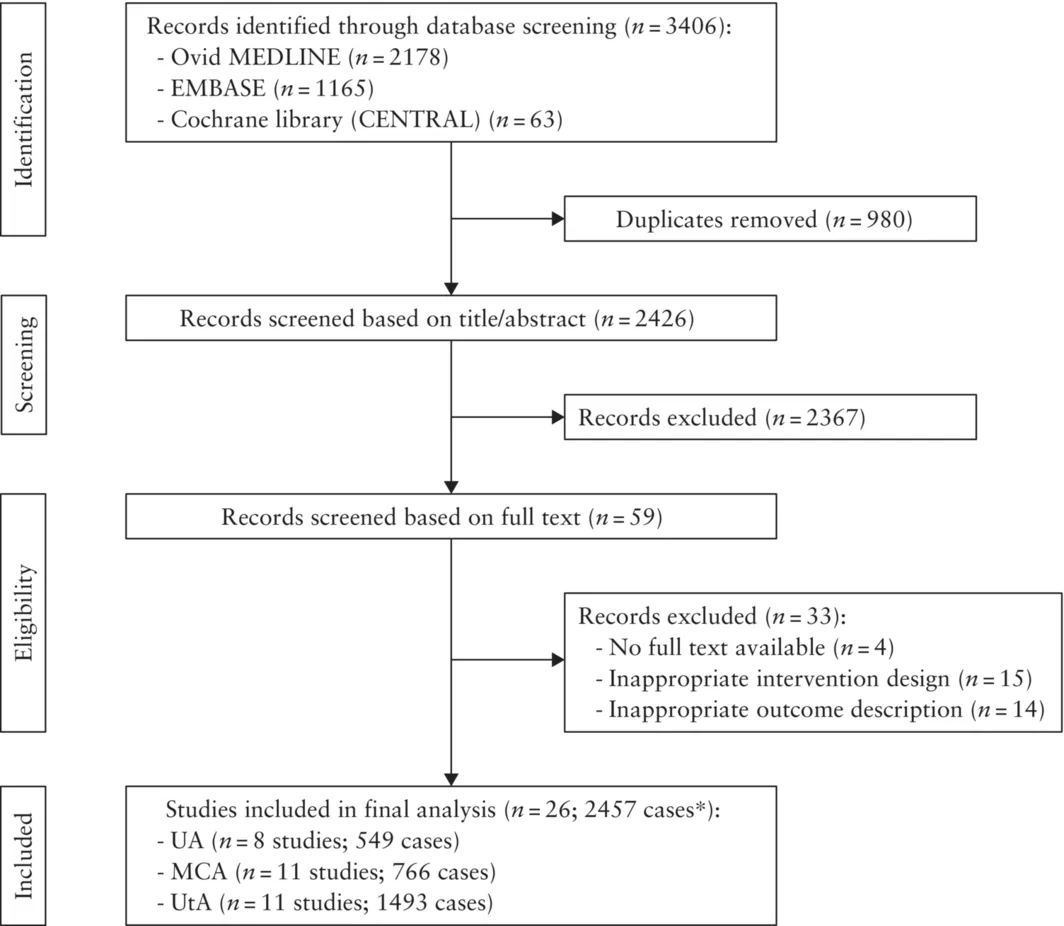
Flowchart summarizing selection of studies for inclusion in systematic review on intra‐ and interobserver variability of fetal and maternal Doppler measurements. In total, 26 studies were included, of which eight analyzed umbilical artery (UA)^11^, ^20^, ^21^, ^28^, ^35^, ^36^, ^38^, ^42^, 11 analyzed middle cerebral artery (MCA)^11^, ^19^, ^20^, ^24^, ^26^, ^28^, ^29^, ^34^, ^37^, ^39^, ^41^ and 11 analyzed uterine artery (UtA)^10^, ^23^, ^25^, ^27^, ^28^, ^30^, ^31^, ^32^, ^33^, ^36^, ^40^ Doppler measurement variability. *Several patients underwent analysis of more than one artery.

Our search identified 3406 records that were potentially eligible for inclusion. Removal of duplicates left 2426 records that underwent screening by title and abstract, of which 59 articles underwent full‐text screening. Of these, 26 studies^10^, ^11^, ^19^, ^20^, ^21^, ^22^, ^23^, ^24^, ^25^, ^26^, ^27^, ^28^, ^29^, ^30^, ^31^, ^32^, ^33^, ^34^, ^35^, ^36^, ^37^, ^38^, ^39^, ^40^, ^41^, ^42^ were identified for inclusion in the final analysis. A flowchart summarizing screening and inclusion of studies in the analysis is presented in Figure 1.

### Study and model characteristics

The 26 included studies (2457 patients) comprised 25 prospective cohort studies and one randomized controlled trial^24^. However, we considered the randomized controlled trial as a prospective cohort study for the purposes of this review, as we used pooled data from all enrolled participants regardless of randomization. An extensive list of study characteristics and results is given in Tables S2 and S3. In total, 1154 patients were included for intraobserver ICC analyses and 1108 patients for interobserver ICC analyses (Table S2).

### Umbilical artery

**Figure 2:**
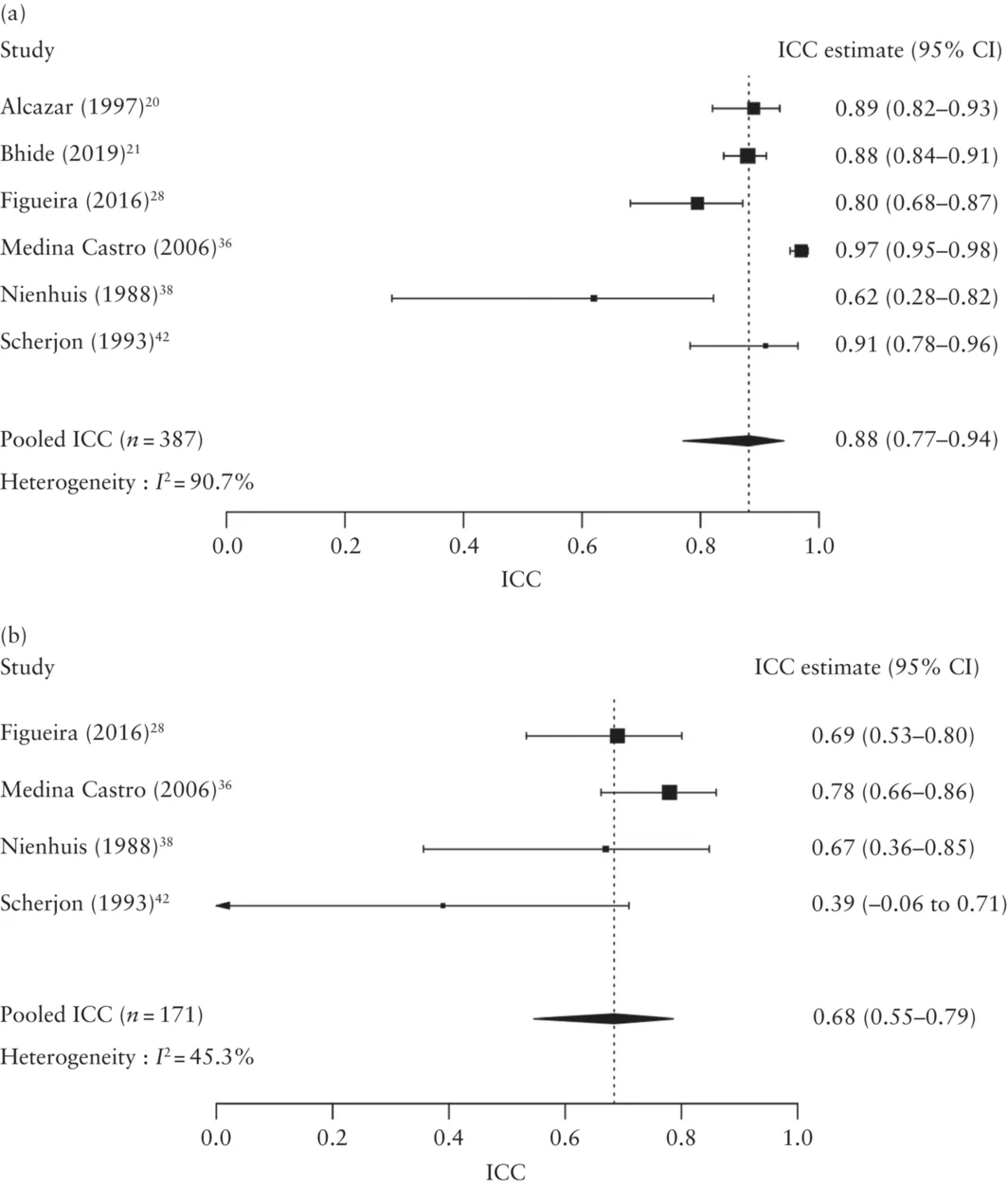
Forest plots showing intraclass correlation coefficients (ICCs) for intra‐ (a) and interobserver (b) variability of umbilical artery pulsatility index (UA‐PI) Doppler measurements. Only first author is given for each study.

Six studies reported intraobserver ICC values for the UA‐PI, involving 387 patients^20^, ^21^, ^28^, ^36^, ^38^, ^42^ (Table 2). The pooled ICC for UA‐PI intraobserver variability was 0.88 (95% CI, 0.77–0.94), indicating poor reproducibility (Figure 2a). According to the TRUST criteria presented in Table 1, the intraobserver reproducibility for UA‐PI ranged from very poor (ICC, 0.62), reported by Nienhuis *et al*.^38^, to good (ICC, 0.97), reported by Medina Castro *et al*.^36^. There were notable differences between the authors' classifications of the variability of UA Doppler measurements and those according to the TRUST criteria (Table 2).

**Table 2:** Intra‐ and interobserver variability of umbilical artery pulsatility index Doppler measurements in included studies, showing reported intraclass correlation coefficients (ICCs), with each study's classification of their findings, and corresponding classification according to True Reproducibility of Ultrasound Techniques (TRUST) criteria

Study	Patients (*n*)	GA (weeks)*, †	ICC‡	Original classification	TRUST classification
Intraobserver variability					
Alcazar (1997)^20^	58	10 + 2 (7 + 4 to 13)*	0.89	Acceptable	Poor
Bhide (2019)^21^	158	32 + 6 (28 + 6 to 36 + 5)†	0.884 (0.814–0.915)	High	Poor
Figueira (2016)^28^	63	28 + 3 (16–41)*	0.795 (0.746–0.835)	Relatively high	Poor
Medina Castro (2006)^36^	65	(20–40)	0.97 (0.93–0.98)	NS	Good
Nienhuis (1988)^38^	23	(18–40)	0.62	Relatively good	Very poor
Scherjon (1993)^42^	20	35 (29–42)†	0.91	Relatively high	Moderate
Interobserver variability					
Figueira (2016)^28^	63	28 + 3 (16–41)*	0.692 (0.590–0.772)	Relatively high	Very poor
Medina Castro (2006)^36^	65	(20–40)	0.78 (0.59–0.88)	NS	Poor
Nienhuis (1988)^38^	23	(18–40)	0.67	Relatively good	Very poor
Scherjon (1993)^42^	20	32 (29–39)†	0.39	Relatively high	Very poor

Only first author of study is specified. Gestational age (GA) is presented as

* mean (range),

† median (range) or (range).

‡ ICC is presented with 95% CI when reported by the original study. NS, not stated.

The interobserver variability of UA‐PI was reported in four studies, involving 171 patients^28^, ^36^, ^38^, ^42^ (Table 2). The pooled ICC for interobserver variability of UA‐PI was 0.68 (95% CI, 0.55–0.79), indicating very poor reproducibility (Figure 2b). The interobserver ICC values in individual studies ranged from 0.39 (very poor), reported by Scherjon *et al*.^42^, to 0.78 (poor), reported by Medina Castro *et al*.^36^.

**Figure 3:**
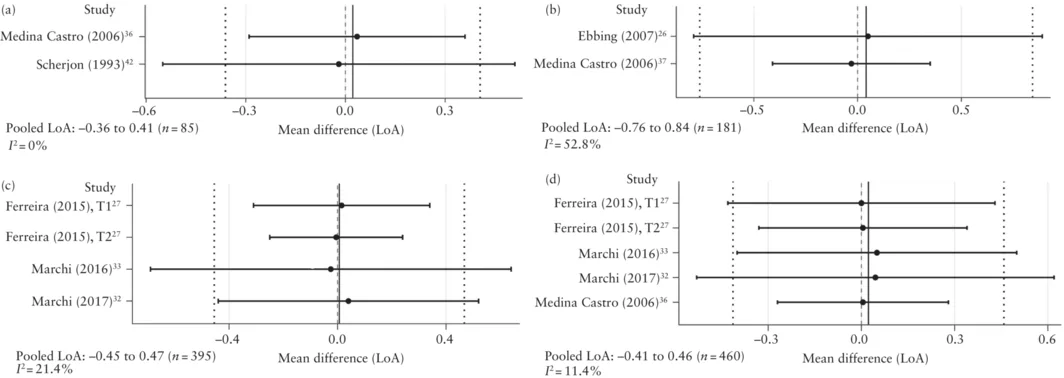
Forest plots of limits of agreement (LoA) for intra‐ and interobserver pulsatility index (PI) Doppler measurements. (a) Interobserver LoA for umbilical artery PI; (b) interobserver LoA for middle cerebral artery PI; and (c,d) intraobserver (c) and interobserver (d) LoA for uterine artery PI. Error bars show 95% LoA (mean ± (1.96 × SD)). Solid vertical lines are pooled mean and dotted lines are pooled LoA.

Three studies reported on LoA for UA‐PI, including 187 patients^11^, ^36^, ^42^ (Table 3). Only one study^11^ reported results on intraobserver variability. The two remaining studies reported on interobserver variability. Therefore, no formal meta‐analysis could be performed. A forest plot was created of the interobserver data, for visual purposes only (Figure 3a).

**Table 3:** Limits of agreement (LoA) from included studies on intraobserver and interobserver variability of umbilical artery pulsatility index (PI), middle cerebral artery PI and uterine artery PI

Study	Intra‐/ interobserver	Patients (*n*)	Lower LoA	Upper LoA	LoA width
Umbilical artery PI					
Segev (2022)^11^	Intraobserver	102	−0.23	0.24	0.47
Medina Castro (2006)^36^	Interobserver	65	−0.29	0.36	0.65
Scherjon (1993)^42^	Interobserver	20	−0.55	0.51	1.06
Middle cerebral artery PI					
Ebbing (2007)^26^	Intraobserver	161	−0.24	0.24	0.48
Medina Castro (2006)^37^	Interobserver	20	−0.41	0.35	0.76
Ebbing (2007)^26^	Interobserver	161	−0.79	0.89	1.68
Uterine artery PI					
Ferreira (2015)^27^: T1	Intraobserver	97	−0.31	0.34	0.65
Ferreira (2015)^27^: T2	Intraobserver	96	−0.25	0.24	0.49
Marchi (2017)^32^	Intraobserver	101	−0.44	0.52	0.96
Marchi (2016)^33^	Intraobserver	101	−0.69	0.64	1.33
Ferreira (2015)^27^: T1	Interobserver	97	−0.43	0.43	0.86
Ferreira (2015)^27^: T2	Interobserver	96	−0.33	0.34	0.67
Marchi (2017)^32^	Interobserver	101	−0.53	0.62	1.15
Marchi (2016)^33^	Interobserver	101	−0.40	0.50	0.90
Medina Castro (2006)^36^	Interobserver	65	−0.27	0.28	0.55

Only first author of each study is given. For Ferreira (2015), only transabdominal values were used. T1, first trimester; T2, second trimester.

Only one study, by Segev *et al*.^11^, reported the Cohen's kappa statistical test for intraobserver variability of UA‐PI, with a reported value of 0.8407 (95% CI, 0.776–0.838) (point estimate outside stated 95% CI; values reported as published in original study). No formal meta‐analysis was performed. Similarly, only one study, by Martinez *et al*.^35^, reported the Lin's CCC statistical test for UA‐PI, with a reported intraobserver value of 0.911 (95% CI, 0.76–0.96) and an interobserver value of 0.623 (95% CI, 0.45–0.87). No formal meta‐analysis was performed.

### Middle cerebral artery

**Figure 4:**
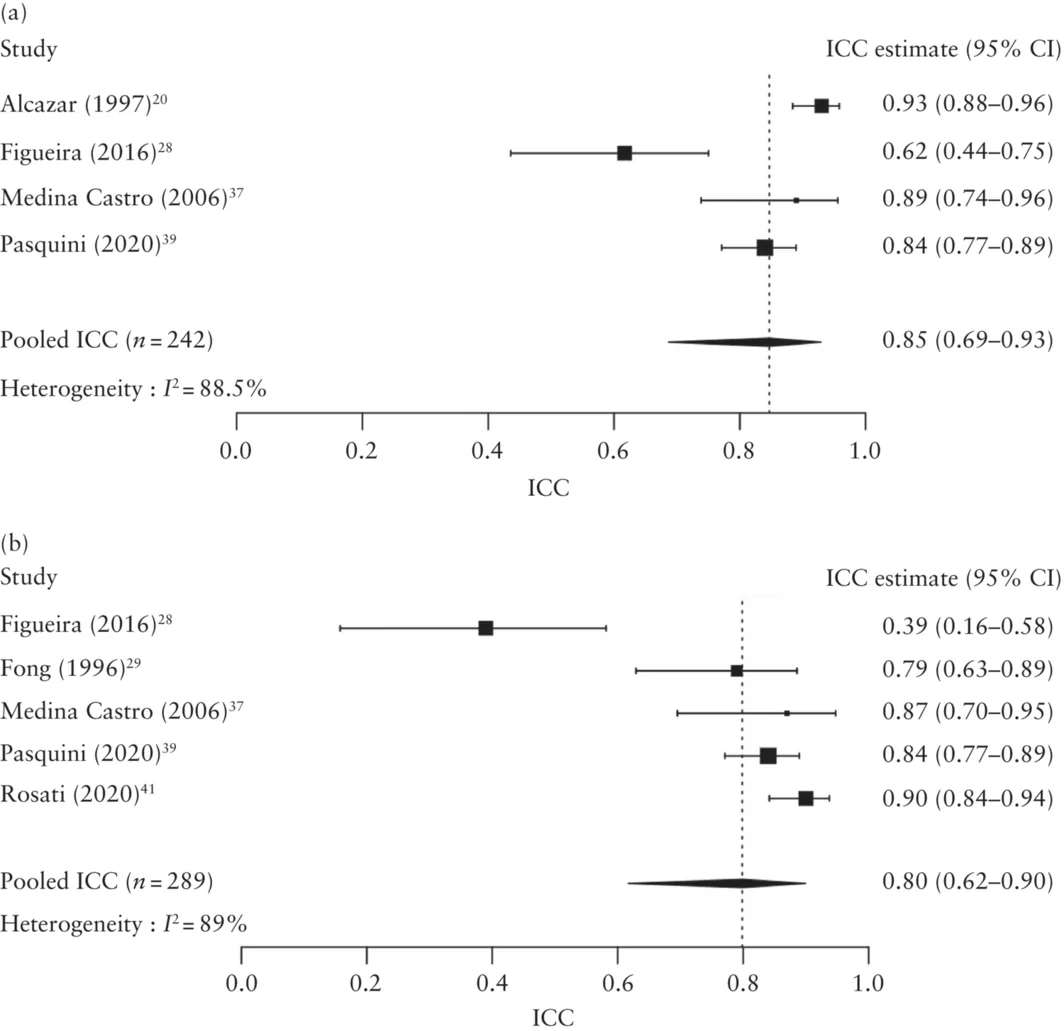
Forest plots showing intraclass correlation coefficients (ICCs) for intra‐ (a) and interobserver (b) variability of middle cerebral artery pulsatility index (MCA‐PI) Doppler measurements. Only first author is given for each study.

Four studies reported the intraobserver variability of MCA‐PI measurements, encompassing a total of 242 patients^20^, ^28^, ^37^, ^39^ (Table 4). The pooled ICC for MCA‐PI intraobserver variability was 0.85 (95% CI, 0.69–0.93), indicating poor reproducibility (Figure 4a). The ICC values ranged from 0.62, reported by Figueira *et al*.^28^, to 0.93, reported by Alcázar^20^, indicating very poor to moderate reproducibility across studies according to the TRUST criteria (Table 4).

**Table 4:** Intra‐ and interobserver variability of middle cerebral artery pulsatility index Doppler measurements in included studies, showing reported intraclass correlation coefficients (ICCs), with each study's classification of their findings, and corresponding classification according to True Reproducibility of Ultrasound Techniques (TRUST) criteria

Study	Patients (*n*)	GA (weeks)*, †	ICC‡	Original classification	TRUST classification
Intraobserver variability					
Alcazar (1997)^20^	58	10 + 2 (7 + 4 to 13)*	0.93	Acceptable	Moderate
Figueira (2016)^28^	63	28 + 3 (16–41)*	0.617 (0.538–0.685)	Relatively high	Very poor
Medina Castro (2006)^37^	20	(20–40)	0.89 (0.65–0.97)	NS	Poor
Pasquini (2020)^39^	101	34 + 3 (30 + 0 to 40 + 0)†	0.84 (0.76–0.89)	Poor	Poor
Interobserver variability					
Figueira (2016)^28^	63	28 + 3 (16–41)*	0.391 (0.234–0.527)	Relatively high	Very poor
Fong (1996)^29^	38	31 + 4 (25–39)*	0.79	Substantial agreement	Poor
Medina Castro (2006)^37^	20	(20–40)	0.87 (0.63–0.93)	NS	Poor
Pasquini (2020)^39^	101	34 + 3 (30 + 0 to 40 + 0)†	0.84 (0.75–0.89)	Poor	Poor
Rosati (2020)^41^	67	40 + 0 (40 + 0 to 40 + 6)* and 41 + 3 (41 + 0 to 41 + 6)*	0.90 (0.85–0.94)	Good	Poor

Only first author of each study is given. Gestational age (GA) is presented as

* mean (range),

† median (range) or (range).

‡ ICC is presented with 95% CI when reported by the original study. NS, not stated.

The interobserver variability of MCA‐PI measurements was reported in five studies, involving a total of 289 patients^28^, ^29^, ^37^, ^39^, ^41^ (Table 4). The pooled ICC for MCA‐PI interobserver variability was 0.80 (95% CI, 0.62–0.90), indicating poor reproducibility (Figure 4b). The ICC values ranged from 0.39, reported by Figueira *et al*.^28^, to 0.90, reported by Rosati *et al*.^41^ (Table 4).

Two studies reported on the LoA for MCA‐PI measurements, with one study reporting both intra‐ and interobserver variability^26^ and one study reporting only interobserver variability^37^, including a total of 181 patients (Table 3). Because of the low number of separate studies on this subject, no formal meta‐analysis could be performed. A forest plot was created of the interobserver data, for visual purposes only (Figure 3b).

The intraobserver variability of MCA peak systolic velocity (PSV) measurements was reported in three studies, including 99 patients^24^, ^28^, ^34^. The study of Clark *et al*.^24^ included and separately analyzed only two groups, each of three patients, so it could not be included in the meta‐analysis owing to insufficient numbers. The pooled estimate of the intraobserver ICC values for the remaining two studies was 0.95 (95% CI, 0.76–0.99) and a visual representation of the variability of MCA‐PSV in these studies is shown in Figure S2a.

Four studies also reported interobserver variability of MCA‐PSV measurements, including a total of 251 patients^19^, ^28^, ^29^, ^34^. The pooled estimate of the ICC values was 0.93 (95% CI, 0.82–0.97) (Figure S2b). There were some differences between the authors' classifications of the variability of MCA‐PI measurements in these studies and those according to the TRUST criteria (Table 4).

Only two studies reported Cohen's kappa values for MCA‐PI^11^, ^39^. Because of the low number of studies, no formal meta‐analysis was performed^43^. Segev *et al*.^11^ reported an intraobserver value of 0.864 (95% CI, 0.808–0.904), while Pasquini *et al*.^39^ presented an intraobserver value of 0.59 and an interobserver value of 0.42.

### Uterine artery

**Figure 5:**
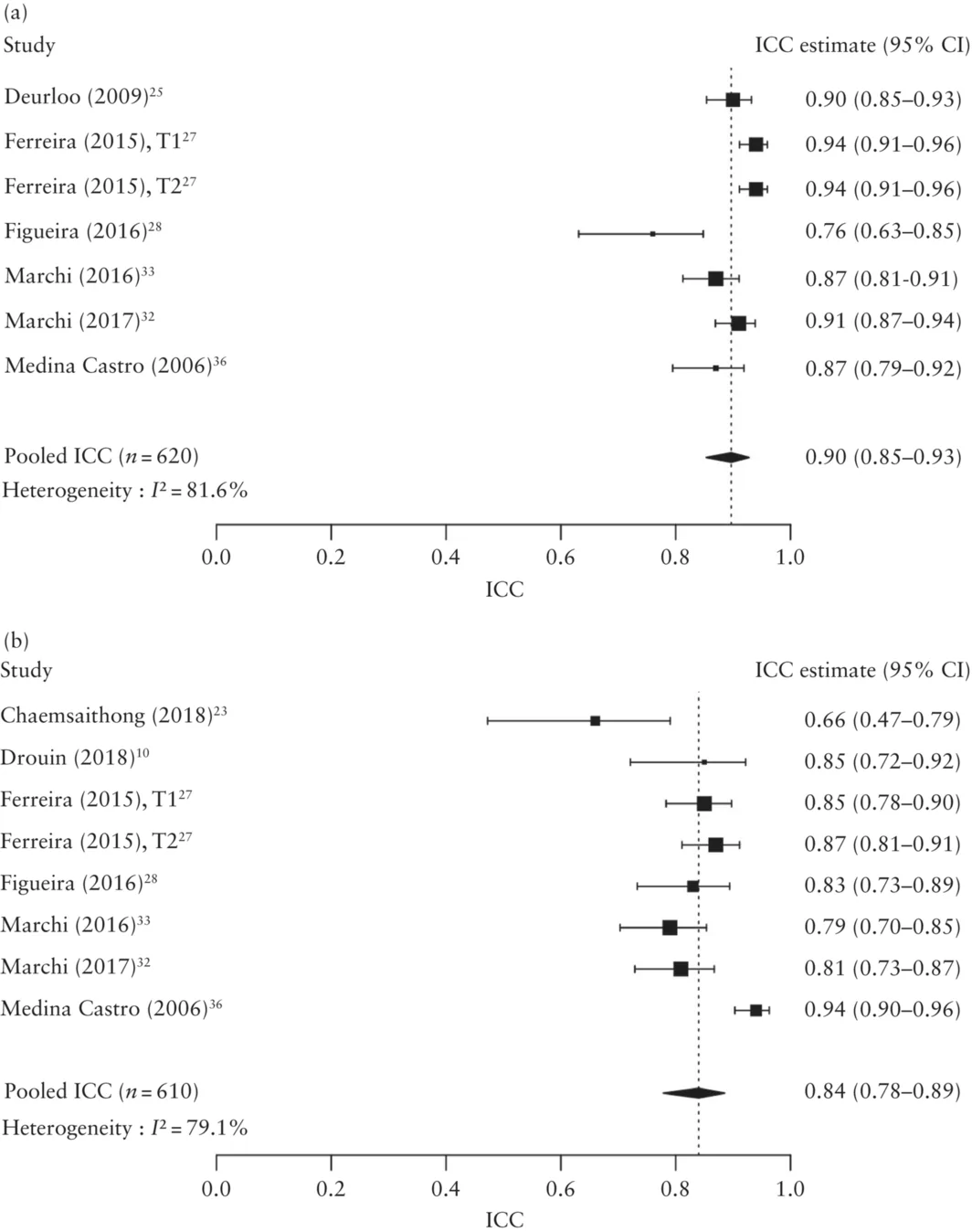
Forest plots showing intraclass correlation coefficients (ICCs) for intra‐ (a) and interobserver (b) variability of uterine artery pulsatility index (UtA‐PI) Doppler measurements. Only first author is given for each study.

The intraobserver variability of UtA‐PI Doppler measurements was reported in nine studies, involving a total of 738 patients^25^, ^27^, ^28^, ^30^, ^31^, ^32^, ^33^, ^36^, ^40^, and intraobserver ICC values of mean UtA‐PI measurements were reported by six studies, including 620 patients^25^, ^27^, ^28^, ^32^, ^33^, ^36^ (Table 5). ICC values of mean UtA‐PI ranged widely across studies, from 0.76, reported by Figueira *et al*.^28^, to 0.94, reported by Ferreira *et al*.^27^. The pooled ICC for intraobserver variability of mean UtA‐PI was 0.90 (95% CI, 0.85–0.93), indicating poor reproducibility (Figure 5a). Three studies reported ICCs for the right and left UtA separately^30^, ^31^, ^40^. ICC values of the right UtA ranged from 0.77^31^ to 0.79^40^. ICC values of the left UtA ranged from 0.50^40^ to 0.87^31^ (Table 5). There were some differences in classification of the variability of UtA‐PI measurements by the authors and according to the TRUST criteria (Table 5).

**Table 5:** Intra‐ and interobserver variability of uterine artery pulsatility index Doppler measurements in included studies, showing reported intraclass correlation coefficients (ICCs), with each study's classification of their findings, and corresponding classification according to True Reproducibility of Ultrasound Techniques (TRUST) criteria

Study	Patients (*n*)	GA (weeks)*, †	ICC‡	Original classification	TRUST classification
Intraobserver variability					
Deurloo (2009)^25^	97	(11–24)	0.90	Good	Poor
Ferreira (2015)^27^: T1	97	12 + 5 (11 to 13 + 6)*	0.94 (0.91–0.96)	Moderate	Moderate
Ferreira (2015)^27^: T2	96	22 + 4 (20–26)*	0.94 (0.91–0.96)	Moderate	Moderate
Figueira (2016)^28^	63	28 + 3 (16–41)*	0.757 (0.701–0.804)	Relatively high	Poor
Hollis (2001)^30^	63	(10–14)	Right: 0.78 (0.66–0.86) Left: 0.80 (0.68–0.87)	Poor	Poor
Irion (1996)^31^	23	18 + 1 and 26 + 6 (18–26)*	Right: 0.77 Left: 0.87	NS	Poor
Marchi (2017)^32^	101	34 + 3 (30–40)†	0.912 (0.882–0.934)	Moderate	Moderate
Marchi (2016)^33^	101	12 + 5 (11 + 0 to 13 + 6)*	0.87	Not satisfactory	Poor
Medina Castro (2006)^36^	65	(20–40)	0.87 (0.74–0.93)	NS	Poor
Ridding (2014)^40^	32	(11 + 0 to 13 + 6)	Right: 0.79 (0.58–0.90) Left: 0.50 (0.16–0.74)	Almost perfect	Right: poor Left: very poor
Interobserver variability					
Chaemsaithong (2018)^23^	52	12 + 3 (11 + 3 to 13 + 5)†	0.66 (0.40–0.80) Right: 0.38 (−0.08 to 0.64) Left: 0.72 (0.51–0.84)	Strong	Very poor
Drouin (2018)^10^	35	12 + 4 (11 + 0 to 13 + 6)*	0.854 (0.744–0.921)	Almost perfect	Poor
Ferreira (2015)^27^: T1	97	12 + 5 (11 + 0 to 13 + 6)*	0.85 (0.78–0.90)	Poor	Poor
Ferreira (2015)^27^: T2	96	22 + 4 (20–26)*	0.87 (0.81–0.91)	Poor	Poor
Figueira (2016)^28^	63	28 + 3 (16–41)*	0.826 (0.761–0.874)	Relatively high	Poor
Hollis (2001)^30^	47	(10–14)	Right: 0.60 Left: 0.58	Poor	Very poor
Marchi (2017)^32^	101	34 + 3 (30 to 40)†	0.809 (0.744–0.858)	Poor	Poor
Marchi (2016)^33^	101	12 + 5 (11 + 0 to 13 + 6)*	0.79	Not satisfactory	Poor
Medina Castro (2006)^36^	65	(20–40)	0.94 (0.88–0.97)	NS	Moderate
Ridding (2014)^40^	26	(11 + 0 to 13 + 6)	Right: 0.93 (0.86–0.96) Left: 0.88 (0.72–0.94)	Almost perfect	Right: moderate Left: poor

Only first author of each study is given. Gestational age (GA) is presented as

* mean (range),

† median (range) or (range).

‡ ICC is presented with 95% CI when reported by the original study. For Ferreira (2015), only transabdominal values were used. NS, not stated; T1, first trimester; T2, second trimester.

The interobserver variability of mean UtA‐PI was reported in seven studies, involving a total of 610 patients^10^, ^23^, ^27^, ^28^, ^32^, ^33^, ^36^ (Table 5). The pooled ICC for UtA interobserver variability was 0.84 (95% CI, 0.78–0.89), indicating poor reproducibility (Figure 5b). The ICC values ranged from 0.66, reported by Chaemsaithong *et al*.^23^, to 0.94, reported by Medina Castro *et al*.^36^ (Table 5).

Three studies reported intraobserver and interobserver CCC data for mean UtA‐PI^27^, ^32^, ^33^, involving a total of 395 patients. For intraobserver variability, the pooled CCC was 0.87 (95% CI, 0.72–0.95) (Figure S3a). For interobserver variability, the pooled CCC was 0.76 (95% CI, 0.58–0.87) (Figure S3b). Both of these values would be considered poor according to the TRUST criteria.

The LoA for intraobserver variability for UtA‐PI measurement were reported in three separate studies, including 395 patients in total^27^, ^32^, ^33^ (Table 3). Meta‐analysis showed pooled LoA ranging from –0.45 to 0.47 (Figure 3c). The pooled mean difference was 0.92, which reflects poor measurement consistency.

For the LoA for interobserver variability for UtA‐PI measurement, four separate studies included a total of 460 patients^27^, ^32^, ^33^, ^36^ (Table 3). The meta‐analysis showed pooled LoA of –0.41 to 0.46, with a mean difference of 0.87 (Figure 3d), which also suggests poor reproducibility.

Only one study, by Irion *et al*.^31^, reported Cohen's kappa for UtA‐PI, with values of 0.74 (95% CI, 0.64–0.83) at 18 weeks and 0.72 (95% CI, 0.64–0.80) at 26 weeks. These values would be considered good according to the TRUST criteria. Because only one study was included, no formal meta‐analysis was performed.

### Cerebroplacental ratio

Only one study, by Bhide *et al*.^22^, reported an intraobserver ICC value for the cerebroplacental ratio (0.884 (95% CI, 0.814–0.915)). One other study, by Segev *et al*.^11^, reported a Cohen's kappa value for intraobserver variability for cerebroplacental ratio measurement of 0.856 (95% CI, 0.797–0.899). Because only one study per statistical analysis was included, no formal meta‐analyses were performed.

### Sensitivity analyses

Excluding older studies that used relatively old ultrasound equipment resulted in the exclusion of 15 of the 26 studies. Analyses were performed in the remaining 11 studies^10^, ^11^, ^21^, ^22^, ^23^, ^27^, ^28^, ^32^, ^33^, ^39^, ^41^. For the UA, only two studies^21^, ^28^ assessing intraobserver variability and one study^28^ assessing interobserver variability remained, precluding calculation of pooled ICC estimates. Similarly, for the MCA, only two studies remained that assessed intraobserver variability^28^, ^39^. For interobserver variability of the MCA‐PI, three studies were available^28^, ^39^, ^41^, yielding a pooled ICC of 0.78 (95% CI, 0.39–0.93) (Figure S4a). For UtA‐PI, the pooled intraobserver ICC was 0.90 (95% CI, 0.84–0.94)^27^, ^28^, ^32^, ^33^ and the pooled interobserver ICC was 0.82 (95% CI, 0.77–0.85)^10^, ^23^, ^27^, ^28^, ^32^, ^33^ (Figure S4b,c).

### Risk of bias and applicability

Most studies had a moderate overall risk of bias, defined as ‘doubtful’ by COSMIN (Figure S1). One study, by Medina Castro *et al*.^37^, excluded cases with an ICC reproducibility value below 0.80. Although these authors reported that observers were standardized to achieve an ICC greater than 0.80, the method used to calculate this threshold was not specified. If this study were to be excluded from the analysis, the pooled estimate of the ICC values for MCA‐PI would be 0.83 (95% CI, 0.59–0.94) for intraobserver variability, and 0.78 (95% CI, 0.54–0.90) for interobserver variability (Figure S5).

## DISCUSSION

In this systematic review, we synthesized evidence from 26 studies evaluating the intra‐ and interobserver variability of Doppler ultrasound measurements for the UA, MCA and UtA. We observed considerable variation in the reported results of observer variability across studies. When benchmarked against the TRUST criteria, the overall reproducibility results were generally classified as very poor to moderate. For UA‐PI ICC, the pooled intra‐ and interobserver reproducibility were classified as poor and very poor, respectively. For the MCA‐PI ICC, the pooled intra‐ and interobserver reproducibility were both poor. Similarly, the pooled ICC for UtA‐PI reproducibility was poor for intra‐ and interobserver variability.

Doppler ultrasound is a valuable tool in obstetric care for assessing the fetal condition, offering both diagnostic and prognostic value. In both low‐ and high‐risk pregnancies, the use of Doppler velocimetry has been shown in randomized controlled trials and meta‐analyses to reduce perinatal complications^1^, ^44^.

The strict TRUST criteria were proposed in 2015 to promote rigorous evaluation of ultrasound studies^7^, ^8^. While several of the studies included in our meta‐analysis classified the variability they observed as ‘moderate’ or ‘acceptable’, or even ‘relatively high’, their classifications were not consistent with those that would have been designated following the TRUST criteria. Instead, observer variability both between and within operators for UA, MCA and UtA Doppler measurements seems widespread. It is important to acknowledge this, regardless of whether its origin lies in biological variation or operator variation, and to take it into account when making clinical decisions.

While the TRUST system provides ultrasound‐specific technical standards and classification thresholds, the broader GRRAS (Guidelines for Reporting Reliability and Agreement Studies) framework, presented by Kottner *et al*.^9^, focuses on transparent reporting and methodological accuracy for any reproducibility study^7^, ^8^. The GRRAS recommend clear reporting of study design and measurement procedures and note that reproducibility estimates of about 0.90–0.95 are necessary when findings are used for individual clinical decisions, whereas lower values may suffice for research purposes. In contrast, the TRUST system does not explicitly distinguish between operator‐dependent variability and physiological, benign variation intrinsic to Doppler measurements^7^, ^8^.

Our findings align with those of previous reviews reporting variability in Doppler measurements. Bruin *et al*.^12^ showed similar results in their systematic review of inter‐ and intraobserver variability of blood‐flow measurements in the DV. A systematic review by Alfirevic *et al*.^45^ similarly highlighted the challenges of achieving consistent UA Doppler readings, particularly in low‐resource settings where operator expertise is limited. Our current review extends this knowledge by providing pooled estimates for both intra‐ and interobserver variability across multiple Doppler parameters, including operators with different levels of experience and offering a more comprehensive evaluation.

Variability in fetal ultrasound measurements can be influenced by several physiological factors, such as maternal BMI, as well as fetal position, bone density and growth variability^46^, ^47^. Also, non‐influential physiological variation of hemodynamic features (e.g. due to fetal movements during examination), amniotic fluid volume and placental position can further add to inconsistent readings^48^, ^49^. Lastly, factors such as operator experience also contribute to variation in results^50^.

We observed considerable variability in reported ICC values for Doppler parameters, which can be attributed to differences in study design, operator experience, measurement protocols, GA at examination and technical factors. Although most studies in our analysis included low‐risk pregnancies, the GA ranged widely from the first trimester to term. For example, Alcázar^20^ and Chaemsaithong *et al*.^23^ focused on first‐trimester assessments, whereas Rosati *et al*.^41^ restricted inclusion to term pregnancies, and others included broad second‐ and third‐trimester ranges. Notably, Alcázar^20^ performed MCA Doppler measurements between 10 and 12 weeks of gestation, which is not common practice. Observer experience also varied considerably, from medical students and early‐career observers to highly experienced sonographers, and this was not reported consistently. In addition, studies differed in technical and temporal context, with older studies probably using less‐advanced ultrasound equipment. Maternal characteristics such as obesity were rarely reported, despite their potential impact on image quality^46^. Such heterogeneity limits direct comparability between studies and should be considered when interpreting the pooled findings.

### Strengths and limitations

This review has several strengths, including a comprehensive search strategy that ensured broad coverage of the literature, the use of robust TRUST criteria to provide objective and consistent classifications, the broad amount of data collected from the studies and the inclusion of multiple Doppler parameters to allow a thorough assessment of observer variability.

However, there are also notable limitations. Heterogeneity in study methodologies, such as differences in Doppler techniques and equipment and the description of variability methods, made comparisons challenging. Additionally, incomplete reporting of variability values and CIs hampered a detailed/complete evaluation of variability in some cases, which precluded the evaluation of some variability tests, such as Cohen's kappa.

### Clinical implications

In routine practice, Doppler measurements are interpreted not in isolation, but rather in combination with other clinical parameters, ensuring a comprehensive assessment of fetal wellbeing. When outliers are detected, quality audits of the measurements are performed, allowing for verification and correction, an approach that is not feasible in blinded research settings. Moreover, while Doppler abnormalities may indicate potential concerns, they are not pathognomonic for adverse outcomes and are not used as stand‐alone diagnostic tools in clinical decision‐making. Instead, they contribute to a broader diagnostic framework that incorporates maternal history, fetal biometry and additional fetomaternal monitoring to guide appropriate management^51^. Our results emphasize that there is still ample room for improvement. This includes adopting uniform guidelines for probe placement, angle correction and sample‐gate size, as recommended by international organizations such as the International Society of Ultrasound in Obstetrics and Gynecology^50^, ^52^. Furthermore, it is advised to perform multiple measurements per session, as well as using the technically best measurement and averaging the left and right results in the case of UtA‐PI, and scheduling several sessions, rather than drawing conclusions based on a single result. Additionally, regular operator training and certification programs are essential to minimize variability and improve accuracy. Automated Doppler software with in‐built error‐checking algorithms could also enhance reproducibility and reduce operator variability^53^.

### Implications for future research

Large, multicenter cross‐sectional studies with strict measurement protocols, including standardized probe placement, angle correction and sample‐gate size, are needed to establish how best to improve the reproducibility of Doppler measurements and assess their impact on clinical outcomes. Additionally, we hypothesize that exploring the role of new imaging techniques, such as three‐dimensional power Doppler or artificial intelligence (AI)‐based image analysis, could revolutionize fetal monitoring by enhancing the accuracy of measurements and reducing interobserver variability^54^, ^55^. These new techniques require rigorous evaluation to ensure widespread implementation.

### Conclusions

Our results emphasize that there is room for improvement regarding the reproducibility of Doppler ultrasound in routine obstetric practice. By standardizing ultrasound measurement protocols, advancing technologies such as AI support and employing adequate training programs, as well as obtaining multiple Doppler measurements per session, performing multiple sessions and performing quality audits when outliers are detected, the field could move towards more accurate and reliable fetal assessment.

## Supporting information


**Table S1** Full search strategy details.
**Table S2** Data extraction from included studies that examined intraclass correlation coefficients (ICCs) for intra‐ and interobserver variability of Doppler ultrasound measurements.
**Table S3** Data extraction from all included studies: study characteristics.
**Figure S1** Risk‐of‐bias assessment using COSMIN tool.
**Figure S2** Forest plots showing estimated intraclass correlation coefficients (ICCs) for intra‐ and interobserver variability of fetal middle cerebral artery peak systolic flow.
**Figure S3** Pooled effect sizes of uterine artery pulsatility index: concordance correlation coefficient (CCC) values.
**Figure S4** Sensitivity analysis of pooled effect sizes of middle cerebral artery and uterine artery pulsatility index, excluding studies published earlier than 2015.
**Figure S5** Pooled effect sizes of middle cerebral artery pulsatility index, excluding studies at high risk of bias.

## Data Availability

The data that supports the findings of this study are available in the supplementary material of this article.
