# Supplementary material for: Interobserver and intraobserver variability of fetal and maternal Doppler measurements: systematic review and meta‐analysis
Source: Ultrasound Obstet Gynecol. 2026 Aug 8;68(3):329–40. doi: 10.1002/uog.70287 (PMC13532133; doi:10.1002/uog.70287)
Supplement: Supplementary file 1 [file UOG-68-329-s001.docx]

**Supplementary material**

| **Table S1** Full search details. | | |
| --- | --- | --- |
| **Source: Ovid MEDLINE(R) ALL <1946 to April 15, 2024>** | | |
| **#** | **Search** | **Results** |
| 1 | exp Ultrasonography, Doppler/ or exp Ultrasonography, Prenatal/ | 109792 |
| 2 | (((fetal* or fetus* or maternal* or prenatal*) adj3 doppler*) or doppler*).ab,kf,ti. | 118020 |
| ***3*** | ***or/1-2 [Ultrasonography doppler]*** | ***181609*** |
| 4 | Pregnancy/ | 1010188 |
| 5 | (pregnan* or gestation* or gravid*).ab,kf,ti. | 758341 |
| ***6*** | ***or/4-5 [Pregnancy]*** | ***1226049*** |
| 7 | observer variation/ or "reproducibility of results"/ | 496982 |
| 8 | ((inter* or intra*) adj3 reliab*).ab,kf,ti. | 55730 |
| 9 | (((interclass* or cohen*) adj3 coefficient*) or ICC or bland-altman*).ab,kf,ti. | 54410 |
| ***10*** | ***or/7-9 [inter- and intraobserver variability]*** | ***547829*** |
| ***11*** | ***3 and 6 [Ultrasonography doppler & Pregnancy]*** | ***44820*** |
| ***12*** | ***and/3,6,10 [Ultrasonography doppler & Pregnancy & inter- and intraobserver variability]*** | ***2299*** |
| 13 | exp animal/ not human/ | 5212238 |
| 14 | disease models, animal/ or exp animals, laboratory/ or exp rodentia/ or cattle/ or exp animals, genetically modified/ | 4042628 |
| 15 | ((animal adj3 (model* or experiment* or genetically modified)) or canine or dog or dogs or beagle* or feline or cat or cats or rodent* or rabbit* or mice or mouse or murine* or rat or rats).ab,kf,ti. | 3799262 |
| 16 | or/13-15 [Animal filter] | 6828295 |
| ***17*** | ***12 not 16 [Animals excluded]*** | ***2242*** |
| 18 | case reports/ | 2396575 |
| 19 | (case serie* or case report* case stud*).ab,ti. | 110316 |
| 20 | or/18-19 [Case studies] | 2484852 |
| ***21*** | ***17 not 20 [Case studies excluded]*** | ***2205*** |
| 22 | letter/ | 1249122 |
| 23 | letter*.pt,ti. | 1297354 |
| 24 | or/22-23 [Letters] | 1297354 |
| **25** | **21 not 24 [Letters excluded]** | **2178** |
|  | | |
| **Source: Ovid - Embase Classic+Embase <1947 to 2024 April 16>** | | |
| **#** | **Search** | **Results** |
| 1 | exp doppler ultrasonography/ or exp fetus echography/ | 85501 |
| 2 | (((fetal* or fetus* or maternal* or prenatal*) adj3 doppler*) or doppler*).ab,kw,ti. | 177867 |
| ***3*** | ***or/1-2 [Ultrasonography doppler]*** | ***231677*** |
| 4 | pregnancy/ | 846570 |
| 5 | (pregnan* or gestation* or gravid*).ab,kw,ti. | 1050558 |
| ***6*** | ***or/4-5 [Pregnancy]*** | ***1353753*** |
| 7 | observer variation/ or reproducibility/ or interrater variability/ | 313898 |
| 8 | ((inter* or intra*) adj3 reliab*).ab,kw,ti. | 69973 |
| 9 | (((interclass* or cohen*) adj3 coefficient*) or ICC or bland-altman*).ab,kw,ti. | 77031 |
| ***10*** | ***or/7-9 [inter- and intraobserver variability]*** | ***407417*** |
| ***11*** | ***3 and 6 [Ultrasonography doppler & Pregnancy]*** | ***38638*** |
| ***12*** | ***and/3,6,10 [Ultrasonography doppler & Pregnancy & inter- and intraobserver variability]*** | ***1219*** |
| 13 | exp animal/ not human/ | 6192030 |
| 14 | ((animal adj3 (model* or experiment*)) or canine or dog or dogs or beagle* or feline or cat or cats or rodent* or rabbit* or mice or mouse or murine* or rat or rats).ab,kw,ti. | 4959775 |
| 15 | or/13-14 [Animal filter] | 7701251 |
| ***16*** | ***12 not 15 [Animals excluded]*** | ***1185*** |
| 17 | case report/ | 3090241 |
| 18 | (case serie* or case report* case stud*).ab,ti. | 156060 |
| 19 | or/17-18 [Case studies] | 3210777 |
| ***20*** | ***16 not 19 [Case studies excluded]*** | ***1169*** |
| 21 | letter/ | 1236423 |
| 22 | letter*.pt,ti. | 1362171 |
| 23 | or/21-22 [Letters] | 1369248 |
| **24** | **20 not 23 [Letters excluded]** | **1165** |
|  | | |
| **Source: Cochrane Library (CENTRAL) <1947 to 2024 April 16>** | | |
| **#** | **Search** | **Results** |
| #1 | MeSH descriptor: [Ultrasonography, Doppler] explode all trees | 3628 |
| #2 | MeSH descriptor: [Ultrasonography, Prenatal] explode all trees | 800 |
| #3 | (((fetal*or fetus* or maternal* or prenatal*) NEAR/3 doppler*) or doppler*):ab,kw,ti | 13664 |
| ***#4*** | ***#1 OR #2 OR #3*** | ***14244*** |
| #5 | MeSH descriptor: [Pregnancy] this term only | 33306 |
| #6 | (pregnan* or gestation* or gravid*):ab,kw,ti | 100461 |
| ***#7*** | ***#5 OR #6*** | ***100461*** |
| #8 | MeSH descriptor: [Observer Variation] this term only | 2688 |
| #9 | MeSH descriptor: [Reproducibility of Results] explode all trees | 16556 |
| #10 | ((inter* or intra*) NEAR/3 reliab*):ab,kw,ti | 5015 |
| #11 | (((interclass* or cohen*) NEAR/3 coefficient*) or ICC or bland-altman*):ab,kw,ti | 3838 |
| ***#12*** | ***#8 OR #9 OR #10 OR #11*** | ***23563*** |
| ***#13*** | ***#4 AND #7*** | ***1863*** |
| **#14** | **#4 AND #7 AND #12** | **63** |
| #15 | #14 in Cochrane Reviews | 1 |
| #16 | #14 in Trials | 62 |

| **Table S2** Data extraction from included studies that examined intraclass correlation coefficients (ICC) for intra- and interobserver variability of Doppler ultrasound measurements | | | | | | | | | |
| --- | --- | --- | --- | --- | --- | --- | --- | --- | --- |
| **Study** | **N** | **Artery** | **Intra-observer variability of ICC with 95% CI (if applicable)** | | | | **Inter- observer variability of ICC with 95% CI (if applicable)** | | |
|  | | | **UA-PI** | MCA-PI **/ MCA-PSV** | **UtA-PI** | **CPR** | **UA-PI** | MCA-PI **/ MCA-PSV** | **UtA-PI** |
| **Abel 2003**^19^ | 120 | MCA | - | - | - | - | - | **0.94** | - |
| **Alcazar 1997**^20^ | 58 | UA MCA | 0.89 | 0.93 | - | - | - | - | - |
| **Bhide 2019**^22^ | 158 | CPR | - | - | - | 0.569  (0.410 – 0.685) | - | - | - |
| **Bhide 2019**^21^ | 158 | UA | 0.884  (0.814-0.915) | - | - | - | - | - | - |
| **Chaemsaithong 2018**^23^ | 52 | UtA | - | - |  | - | - | - | Mean: 0.66  (0.40–0.80)  Right: 0.38  (-0.08–0.64)  Left: 0.72  (0.51–0.84) |
| **Clark 2009 Group A**^24^ | 3 | MCA |  | **0.84  (0.59–0.96)** |  |  |  | **0.94  (0.81–0.98)** |  |
| **Clark 2009 Group B**^24^ | 3 | MCA |  | **0.79  (0.49–0.94)** |  |  |  | **0.92  (0.84–0.98)** |  |
| **Deurloo 2009**^25^ | 97 | UtA | - | - | 0.90 | - | - | - | - |
| **Drouin 2018**^10^ | 35 | UtA | - | - | - | - | - | - | 0.854  (0.744–0.921) |
| **Ferreira 2015 T1**^27^ | 97 | UtA | - | - | 0.94  (0.91–0.96) | - | - | - | 0.85  (0.78–0.90) |
| **Ferreira 2015 T2**^27^ | 96 | UtA | - | - | 0.94  (0.91–0.96) | - | - | - | 0.87  (0.81–0.91) |
| **Figueira 2016**^28^ | 63 | UA  MCA UtA | 0.795  (0.746–0.835) | 0.617 (0.538 – 0.685)   **0.891 (0.864 – 0.931)** | 0.757  (0.701–0.804) | - | 0.692  (0.590–0.772) | 0.391  (0.234–0.527)  **0.926  (0.896–0.947)** | 0.826  (0.761–0.874) |
| **Fong 1996**^29^ | 38 | MCA | - | - | - | - | - | 0.79 **0.75** | - |
| **Hollis 2001**^30^ | 63 (intra)  47 (inter) | UtA | - | - | Right: 0.78  (0.66–0.86)  Left: 0.80  (0.68–0.87) | - | - | - | Right: 0.60  Left: 0.58 |
| **Irion 1996**^31^ | 23 | UtA | - | - | Right: 0.77  Left: 0.87 | - | - | - |  |
| **Marchi 2017**^32^ | 101 | UtA | - | - | 0.912  (0.882‐0.934) | - | - | - | 0.809  (0.744 - 0.858) |
| **Marchi 2016**^33^ | 101 | UtA | - | - | 0.87 | - | - | - | 0.79 |
| **Mari 2005**^34^ | 30 | MCA | - | **0.98** | - | - | - | **0.98** | - |
| **Medina Castro 2006**^37^ | 20 | MCA | - | 0.89  (0.65-0.97) | - | - | - | 0.87  (0.63-0.93) | - |
| **Medina Castro 2006**^36^ | 65 | UA UtA | 0.97  (0.93-0.98) | - | 0.87  (0.74-0.93) | - | 0.78  (0.59-0.88) | - | 0.94  (0.88-0.97) |
| **Nienhuis 1988**^38^ | 23 | UA | 0.62 | - | - | - | 0.67 | - | - |
| **Pasquini 2020**^39^ | 101 | MCA | - | 0.84  (0.76 to 0.89) | - | - | - | 0.84  (0.75 to 0.89) | - |
| **Ridding 2014**^40^ | 32 (intra)  26 (inter) | UtA | - | - | Right: 0.79 (0.58–0.90)  Left: 0.50 (0.16–0.74) | - | - | - | Right: 0.93 (0.86–0.96)  Left: 0.88 (0.72–0.94) |
| **Rosati 2020**^41^ | 67 | MCA | - | - | - | - | - | 0.90  (0.85–0.94) | - |
| **Scherjon 1993**^42^ | 20 | UA | 0.91 | - | - | - | 0.39 | - | - |
| Table S2. Data extraction from included studies on intra- and interobserver variability of Doppler ultrasound measurements: intraclass correlation coefficients (ICC). For UA and Uta, only PI measurements. For MCA, both PI and/or PSV values are presented.  N = number. UA = umbilical artery. MCA = middle cerebral artery. UtA = uterine artery. CPR = cerebroplacental ratio. PI = pulsatility index. PSV = peak systolic flow.  Bold numbers = PSV measurement instead of PI | | | | | | | | | |

| **Table S3** Data extraction from all included studies: study characteristics. | | | | | | | | | | | |
| --- | --- | --- | --- | --- | --- | --- | --- | --- | --- | --- | --- |
| **Study** | **Clinical Setting** | **Inclusion Criteria** | **Population Type** | **Mean Maternal Age (years)** | **BMI** | **GA Range (weeks)** | **Mean or Median GA (weeks)** | **TA/**  **TV** | **Ultrasound machine** | **Sonographer Experience** | **Measurement Approach** |
| **Abel 2003**^19^ ***n* = 120** | Fetal Diagnostic Center at Duke.  University hospital | Singleton pregnancies, normal fetuses, no anomalies. | Low-risk | NS | NS | 16–42 | 27+0 | TA | Acuson 128 XP, Acuson Sequoia or Philips GE Voluson 730 Expert | Experienced. Years not specified | Each sonographer performed full measurement sets per fetus |
| **Alcazar 1997**^20^ ***n* = 58** | Diagnostic Ultrasound Unit. Teaching/ University Hospital | Low-risk, singleton nancies; part of first-trimester workup | Low risk | 29 | NS | 7+4–13 | 10+2 | TV | Philips P700-SE | 3 years | Same sonographer performed all 3 measurements per fetus at different times (before, during, and after the standard ultrasound) |
| **Bhide (CPR) 2019**^22^ ***n* = 158** | St. George’s Hospital, London,  Teaching hospital | Singleton pregnancy, secure dating | Low-risk | NS | NS | 28+6 - 36+5 | Median 32+6 | TA | NS | NS | Each fetus scanned twice by the same examiner |
| **Bhide (UA) 2019**^21^ ***n* = 158** | St. George’s Hospital, London,  Teaching hospital | Singleton pregnancy, secure dating | Low-risk | NS | NS | 28+6 - 36+5 | Median 32+6 | TA | NS | NS | Each fetus scanned twice by the same examiner |
| **Chaemsaithong 2018**^23^ ***n* = 52** | Prince of Wales Hospital, Hong Kong  University Hospital | Singleton pregnancies, first trimester UtA PI measurements | Low-risk | 32.7 years  (19.3–41.9) | 21.5 | 11+3 - 13+5 | Median 12+3 | TA | Phillips | Sonographer A 10 years  Sonographer B >15 years | Doppler assessment consecutively by the two sonographers, both were unaware of each other’s UtA- measurements. |
| **Clark 2009**^24^ ***n* = 6** | Urban tertiary care hospital.  Teaching/ University Hospital | Singleton pregnancies | AGA fetuses of un-complicated pregnancies | NS | 29.7 ± 3.7 | 27–33 | 29+2 ± 2+4 | TA | Acuson Sequoia; Siemens | 13.1 ± 6.3 (3.5 to 20 years) | Sonographers performed 3 sets of MCA, AFI, and DVP measurements on 3 patients, repeated 1 week later with or without specific training to assess intra- and interobserver variability |
| **Deurloo 2009**^25^ ***n* = 97** | Obstetric Department, VUmc. University Hospital | Nulliparous, singleton, uncomplicated pregnancies | Low risk | 32.3 | 22.3 (17-28) | 11 – 24 | NA | TA | Esaote Technos MX | NS | Each artery was measured twice with repositioning; same examiner did all measurements across three gestational windows |
| **Drouin 2018**^10^ ***n* = 35** | NA  University Hospital | Routine first-trimester combined screening | NS | 32.1  (experienced sonographer group),  32.6 (in-experienced sonographer group) | 21.3  (experienced group),  21.0 (in-experienced group) | 11 + 0 - 13 + 6 | 12+4 | TA | Part 1: Toshiba Aplio™;  Part 2: Philips | Experienced sonographer: 11 years; Inexperienced: 6–9 years in obstetric ultrasound, no prior first-trimester UtA-PI experience | Sonographers used randomly ordered sagittal and transverse techniques. In Part 1, they were not blinded to the first technique's results. In Part 2, inexperienced sonographers were also randomized and blinded to each other’s measurements. |
| **Ebbing 2007**^26^ ***n* = 161** | NA  University Hospital | Single pregnancies | Low risk | 29 | 22.9 | 19 - 41 | Median 23.3 (19.3 – 28.4) | TA | Voluson 730 Expert | NS | Each woman was examined three to five times, at 3-5 weeks interval |
| **Ferreira 2015**  **T1**^27^ ***n* = 97** | Sao Paolo UMC Brazil. University and ultrasound school | Singleton pregnancies, normal development | Low-risk | 23.8 ± 5.0 | 26.5 ± 3.0 | 11+0–13+6 | 12.7 ± 2 | TA and TV | GE Voluson E8 | 15 and 3 years (two observers) | Observer A → Observer B → Observer A again |
| **Ferreira 2015**  **T2**^27^ ***n* = 96** | Sao Paolo UMC Brazil. University and ultrasound school | Singleton pregnancies, normal development | Low-risk | 25.0 ± 5.4 | 26.0 ± 3.2 | 20–26 | 22.5 ± 2 | TA and TV | GE Voluson E8 | 15 and 3 years (two observers) | Observer A → Observer B → Observer A again |
| **Figueira 2016**^28^ ***n* = 63** | University of Campinas Medical School.  University hospital | Low-risk pregnancy | Low-risk | 27 | 45% obese | 16 - 41 | 28+3 | TA | Voluson Expert 750, GE Medical Systems or Xario, Toshiba 1 | NS | Examinations were carried out twice, either by the same or two different ultrasonographers, assessing intra- and interobserver variability. |
| **Fong 1996**^29^ ***n* = 38** | Women's College Hospital, University of Toronto.  Teaching/ University Hospital | Singleton pregnancy >24 weeks GA | Both high risk and low risk | NS | NS | 25 - 39 | 31+4 ± 3+4 | TA | Ultramark-9 machine | Two trained observers, years not specified | Two observers measured same vessels within 30 min, blinded to each other’s results; exams recorded and best trace (≥5 waveforms) was used. |
| **Hollis 2001**^30^ ***n* = 110** | Pre-operative Termination Clinics, St George Londen.  Teaching Hospital | Women undergoing termination of pregnancy | NS | 29 (range 17–42) | NS | 10 – 14 | NA | TA | ATL HDI 5000 (5 MHz curvilinear) | 2 trained observers, years not specified | Observer 1 performed two measurements (≥2 min apart), then observer 2 assessed independently |
| **Irion 1996**^31^ ***n* = 665**† | Tertiary care setting.  University hospital | Nulliparous women in pre-eclampsia prediction study | Low risk | 26.7 (SD 4.5) | NS | 18 - 26 | 18+1 (SD 1+1) & 26+6 (SD 1) | TA | Aloka SSD-680 | NS | Observer blinded to clinical data; independent review of images; both uterine arteries assessed after rest |
| **Marchi 2017**^32^ ***n* = 101** | Fetal Medicine Unit, Florence.  University hospital | Routine third trimester scan | Mixed (mostly low risk, SGA fetuses) | 34 (range 18–43) | 26 (range 21–46) | 30 – 40 | Median 34+3 | TA | GE Voluson E8 (2–5 MHz probe) | Both FMF-certified, years not specified | Operators A and B alternated: each did 2 scans per case; blinded and independent acquisitions |
| **Marchi 2016**^33^ ***n* = 101** | Prenatal Diagnosis Centers.  University hospital + screening center | Low-risk, singleton pregnancies, first-trimester screening | Low risk | 31 | 24.1 (mean ±4.2) | 11+0 – 13+6 | 12+5 | TA and TV | GE Voluson E8 and Hitachi Aloka ProSound Alpha | Operator A: >3 years (1000+ measurements); Operator B: 6 months; Operator C: 2 years | Operator A did full set (TA right/left twice, then TV right/left twice), then Operator B or C repeated. Independent, blinded measurements. |
| **Mari 2005**^34^ ***n* = 30** | Two different institutions.  University hospitals | Assessment of intraobserver and interobserver variability. Two separate instititions | AGA fetuses | NS | NS | 14 - 37.5 | 25.8 ± 7+2  &  27+5 ± 5+4 | TA | Acuson Sequoia, HDI 5000, LOGIQ 700 | NS | MCA PSV measured at 3 sites and contralateral origin. Two observer pairs per site; each sampled twice. |
| **Martinez 1995**^35^ ***n* = 60** | Hospital clinic | Routine first trimester examination | NS | 36.8 | NS | 10-13 | 11.4 ±1.81 | TA | Hitachi 525-A | Two observers with comparable extensive experience in untrasound | Two measurements with 5 min interval were performed |
| **Medina Castro (UA, UtA) 2006**^36^ ***n* = 65** | 2 hospitals in Mexico | Singleton, normally growing fetuses | Low-risk | 23 (range 15–37) | NS | 20–40 | NS | TA | Philips ATL-HDI-5000 and Siemens Sonolin E Antares | NS | Each operator repeated the full process for reproducibility |
| **Medina Castro  (MCA) 2006**^37^ ***n* = 20** | Fetal Medicine Department in Mexico.  University hospital | Uncomplicated, singleton pregnancies | Low-risk | 23 (range 15-37) | NS | 20-40 | NS | TA | Sonoline Antares (Siemens) | NS | Pulsatility index obtained by automated analysis of flow velocity waveform over five uniform cycles. Reproducibility was assessed in 20 fetuses |
| **Nienhuis 1988**^38^ ***n* = 23** | University/ Teaching hospital | Uncomplicated singleton pregnancies | Low risk | NS | NS | 18-40 | NS | TA | Pie Data 900 (Pedof) | One with 3 years of experience, one relatively new, and one medical student with several weeks of training | Most skilled examiner scanned first and last; others performed randomly as 2nd or 3rd. Exams lasted ~45 min. Same Doppler equipment and standardized technique used; one examiner processed all data |
| **Pasquini 2020**^39^ ***n* = 101** | Fetal Medicine Unit and Prenatal Diagnosis of Careggi Florence, Italy.  University Hospital | Singleton pregnancies 30+0 - 40+0 weeks | NS | 34 (IQR 28.5–36) | 26 (IQR 24–28) | 30+0 - 40+0 | Median 34+3 (IQR 31+6–36+6) | TA | Voluson E8 ultrasound machine (GE Healthcare Austria GmbH & Co., OG Zipf) | Two experienced operators, both with FMF certificate in Doppler Ultrasound; years not specified | Two operators performed two measurements each, independently and blindly. Second measurement of first operator was done directly after the first one, and the second operator repeated the measurements in the same way after the first operator finished. |
| **Ridding 2014**^40^ ***n* = 58** | Private obstetric ultrasound company | first-trimester aneuploidy screening | Low-risk | 33.5 (range 19–45) | 26.9% obese | 11+0 -13+6 | NS | TA | NS | Experienced, years not specified | Intra-observer: same operator repeated measurements within 30 min. Inter-observer: second blinded operator repeated scan within 30 min |
| **Rosati 2020**^41^ ***n* = 67** | Fondazione Policlinico Universitario A. Gemelli Roma, Italy.  University Hospital | Healthy pregnant women, full term (GA 40 + 0–40 + 6) and late term (GA >41) | Low risk | NS | NS | 40 + 0–40 + 6  &  41 + 0–41 + 6 | 40 & 41+3 | TA | Voluson E8 ultrasound machine | Three independent ultrasonographers, years not specified | Three sonographers independently acquired measurements with manually adjusted insonation angles; >30° excluded. Only final exam per patient was included. |
| **Scherjon 1993**^42^ ***n* = 20** | Tertiary referral hospital | High-risk: suspected SGA or threatened preterm labor, PIH | High risk | NS | NS | Intra-observer: 29 - 42  Inter-observer: 29 - 39 | Intra-observer: median 35  Inter-observer: median 32 | TA | Diasonics DRF 400 with a 2 MHz pulsed Doppler probe | Regular routine obstetrical ultrasound examinations; equally skilled in fetal Dopplers | Observers were unaware of each other’s results and fetuses were investigated in random order; each observer performed the measurement twice in each fetus |
| **Segev 2022**^11^ ***n* = 102** | Tertiary medical center | Routine evaluation | Low risk | NS | NS | 37 - 42 | 40+0 |  | Voluson E6 | One examiner | Flow of MCA and UA were measured three times with 5-10 min interval |
| Table S3. Extraction table study specific parameters. †Irion et al.^31^ included 665 patients for Cohen’s kappa statistical test, of whom 23 were assessed for intraclass correlation coefficient analysis.  BMI= body mass index. GA= gestational age. TA= transabdominal. TV= transvaginal. NS= not specified. AGA= appropriate for gestational age. MCA= middle cerebral artery. AFI= amniotic fluid index. DPV= deepest vertical pocket. UtA= uterine artery. PI= pulsatility index. PSV= peak systolic flow. IQR= interquartile range. SGA= small for gestational age. PIH= pregnancy induces hypertension. | | | | | | | | | | | |

| **Figure S1** Risk-of-bias assessment using COSMIN tool. Two studies performed by Bhide et al. were published in the same year. Ferreira et al. had their study population divided into two groups; T1 = first trimester, T2 = second trimester. | | | | | | | | | |
| --- | --- | --- | --- | --- | --- | --- | --- | --- | --- |
| Study ID | Patients stable | Appropriate time interval | Similar measurement conditions | Blinded administration | Blinded scoring | Other important flaws | Appropriate reliability statistic (e.g. ICC) | | Overall COSMIN reliability RoB |
| **Abel 2003^19^** | VG | VG | VG | D | N/A | A | VG | | D |
| **Alcazar 1997^20^** | VG | VG | VG | D | N/A | A | VG | | D |
| **Bhide (CPR) 2019^22^** | VG | VG | VG | D | N/A | A | VG | | D |
| **Bhide (UA) 2019^21^** | VG | VG | VG | D | N/A | A | VG | | D |
| **Chaemsaithong 2018^23^** | VG | D | VG | VG | VG | D | VG | | D |
| **Clark 2009^24^** | VG | A | VG | A | N/A | A | VG | | A |
| **Deurloo 2009^25^** | VG | VG | VG | A | N/A | A | VG | | D |
| **Drouin 2018^10^** | VG | VG | VG | D | N/A | D | VG | | D |
| **Ebbing 2007^26^** | VG | VG | VG | D | D | VG | VG | | D |
| **Ferreira 2015, T1^27^** | VG | VG | VG | VG | N/A | VG | VG | | VG |
| **Ferreira 2015, T2^27^** | VG | VG | VG | D | N/A | VG | VG | | D |
| **Figueira 2016^28^** | VG | VG | VG | VG | N/A | VG | A | | A |
| **Fong 1996^29^** | VG | VG | VG | A | N/A | VG | VG | | A |
| **Hollis 2001^30^** | D | VG | VG | VG | VG | VG | VG | | D |
| **Irion 1996^31^** | VG | VG | VG | VG | N/A | VG | VG | | VG |
| **Marchi 2017^32^** | VG | VG | A | VG | N/A | VG | VG | | A |
| **Marchi 2016^33^** | VG | VG | VG | A | N/A | VG | VG | | D |
| **Mari 2005^34^** | A | D | VG | VG | N/A | A | VG | | D |
| **Martinez 1995^35^** | D | D | D | D | N/A | D | VG | | D |
| **Medina Castro (UA, UtA) 2006^36^** | D | D | D | D | N/A | D | VG | | D |
| **Medina Castro (MCA) 2006^37^** | VG | D | VG | D | N/A | A | VG | | D |
| **Nienhuis 1988^38^** | VG | VG | VG | VG | N/A | VG | VG | | VG |
| **Pasquini 2020^39^** | VG | VG | VG | VG | N/A | A | VG | | A |
| **Ridding 2014^40^** | VG | D | VG | D | N/A | D | VG | | D |
| **Rosati 2020^41^** | A | D | VG | A | N/A | A | VG | | D |
| **Scherjon 1993^42^** | VG | D | VG | D | N/A | VG | VG | | D |
| **Segev 2022^11^** | VG | VG | VG | D | D | A | VG | | D |
|  |  |  |  |  |  |  |  | |  |
|  | VG = | Very good | A = | Acceptable | D = | Doubtful | N/A = | Not applicable | |

**Figure S2** Intra- and interobserver variability: forest plot of fetal middle cerebral artery peak systolic flow

**Figure S2** (A) Intra- and (B) inter-observer variability of the middle cerebral artery peak systolic flow, presenting estimated ICC values per study and the pooled ICC value. Y-axis show studies with data on particular vessel. X-axis shows ICC value range.
ICC = intraclass correlation coefficient. n = number

**Figure S3** Pooled effect sizes of uterine artery pulsatility index, expressing concordance correlation coefficient values.

**Figure S3** (A) Intra- and (B) inter-observer variability of the uterine artery PI, presenting estimated CCC values per study and the pooled CCC value. Y-axis show studies with data on particular vessel. X-axis shows CCC value range.
CCC= concordance correlation coefficient. PI = pulsatility index. n = number

**Figure S4** Sensitivity analysis of pooled effect sizes of middle cerebral artery pulsatility index (PI) and uterine artery PI, excluding studies published earlier than 2015.

**Figure S4** (A) Inter-observer middle cerebral artery PI variability, (B) intra-observer and (C) interobserver variability of the uterine artery PI with recent studies, presenting estimated ICC values per study and the pooled ICC value. Y-axis show studies with data on particular vessel. X-axis shows ICC value range.
ICC = intraclass correlation coefficient. PI = pulsatility index. n = number

**Figure S5** Pooled effect sizes of middle cerebral artery pulsatility index, excluding critical risk-of-bias studies.

**Figure S5** (A) Intra- and (B) inter-observer variability of the middle cerebral artery PI without the critically high risk of biased studies, presenting effect sizes calculated from original ICC values. Y-axis show studies with data on particular vessel. X-axis show transformed ICC values with the pooled transformed ICC value as reference. ICC = intraclass correlation coefficient. PI = pulsatility index. n = number
